# Tumor interactions with soluble factors and the nervous system

**DOI:** 10.1186/1478-811X-8-21

**Published:** 2010-09-07

**Authors:** Melanie J Voss, Frank Entschladen

**Affiliations:** 1 Institute of Immunology, ZBAF, Witten/Herdecke University, Stockumer Str. 10, 58448 Witten, Germany

## Abstract

In the genomic era of cancer research, the development of metastases has been attributed to mutations in the tumor that enable the cells to migrate. However, gene analyses revealed that primary tumors and metastases were in some cases genetically identical and the question was raised whether metastasis formation might be an inherent feature of certain tumor cells. In contradiction to this view, the last decade of cancer research has brought to light, that tumor cell migration, similar to leukocyte and fibroblast migration, is a highly regulated process. The nervous system plays an important role in this regulation, at least in two respects: firstly, neurotransmitters are known to regulate the migratory activity of tumor cells, and secondly, nerve fibers are used as routes for perineural invasion. We also summarize here the current knowledge on the innervation of tumors. Such a process might establish a neuro-neoplastic synapse, with the close interaction of tumor cells and nerve cells supporting metastasis formation.

## Introduction

In January 1971, the US president Richard Nixon introduced in his State of the Union Address the 'War on Cancer' with the aim to find ways to cure cancer within the next 25 years. Although significant efforts and advances have been made since then, we are still in a war with cancer and far away from a definite victory. With reference to president Nixon's State of the Union Address, Michael Sporn published an article in The Lancet in 1996, in which he reviewed the progress that was made since 1971, and he pointed out the future goals of cancer research [1]. One key statement of this article was: "It is local invasion and distant metastasis that kill rather than excessive cell proliferation *per se*." In fact today over 90 percent of those patients that die from their cancer disease do not die due to the primary tumor but due to the development of metastases. Thus, there is a pressing need of research on how metastases occur, and on ways to prevent or treat this ultimate step in cancer progression.

The end of the previous century was called the genomic era with respect not only to cancer research. Deciphering the human genome was probably the most ambitious project, which actually succeeded in the year 2000 [2]. At this time, genetic models for the development of cancer have been established which have delivered a molecular fundament for the understanding of processes in cancer cells. One of the earliest and most famous is the model by Fearon and Vogelstein (1990) which gives a precise line of genetic events that occur during the transformation of normal colon epithelium to a carcinoma [3]. However, this model of colorectal tumorigenesis does not specify mutations that may constitute the step from carcinoma to metastasis. Since then a still ongoing debate was raised to what extent metastasis formation might be genetically determined. Bernards and Weinberg provided a concept that the tendency to metastasize is acquired early in tumorigenesis [4]. This assumption is based on observations that primary tumors are genetically similar or maybe even equal to their metastases. However, some genes have been identified that are associated with metastasis formation. For example, the analysis of the Smad4 gene in colorectal carcinomas showed mutations in 7 percent of the samples of primary invasive carcinoma without distant metastasis, but mutations in 35 percent of the samples of primary invasive carcinoma with distant metastasis [5]. In 2008, MACC1, a yet largely uncharacterized protein with putative adaptor function, was also shown to be a prominent driver of colorectal metastasis [6]. More recently an amplification of the MTDH gene encoding the protein metadherin has been associated with a promotion of metastasis formation in several types of cancer [7].

In addition to this genetically based view there are several arguments for a non-genetic regulation of metastasis formation. One of the first and most interesting studies was on the chemokine stromal cell-derived factor-1 (SDF-1) and its receptor CXCR4. Müller *et al. *showed that breast cancer cells express this and other chemokine receptors [8]. A blockade of CXCR4 resulted in an impaired metastasis to lymph nodes and lungs in SCID mice experiments. This result provides evidence that metastasis formation is not solely genetically based but regulated by soluble signal substances as well. Müller *et al. *drew parallels to the regulation of leukocyte trafficking, for which the chemokine system is essential.

Tumor cell migration is an essential part of the metastasis cascade, at least in two steps [9]. Firstly, the tumor cells have to emigrate from the primary tumor and enter the site of dissemination, either hematogeneous or lymphogeneous, with the lymphogeneous route discussed to be a default pathway for tumors incapable of crossing blood vessel endothelia [10]. Secondly, the tumor cells have to extravasate form the blood stream and enter the tissue beyond. In the last years several signal substances of different classes have been identified that regulate tumor cell migration. Besides the above introduced chemokines, cytokines are important regulators, too. For example, the transforming growth factor-β induces migration in breast carcinoma cells independent of Smad4, whereas the proliferation of epithelial cells is mediated by a pathway involving Smad [11]. This shows with regard to the aforementioned role of Smad4 that both genetic alterations and non-genetic signalling processes can regulate metastasis formation.

## Neurotransmitters in metastasis formation

### G protein-coupled receptors

Chemokines are well known for their function in leukocyte trafficking and have also been shown to play a role in tumor cell migration and metastasis development [12]. They bind to receptors of the G protein-coupled receptor (GPCR) family, an attribute shared with a plethora of neurotransmitters. It thus seems reasonable to assume that neurotransmitters could play a role in the regulation of tumor cell migration or other parts of the metastasis cascade similar to chemokines. In support of this concept, a number of neurotransmitters have been described in the last decade to have such a function, with the catecholaminergic system being best characterized.

### Catecholamines

**Figure 1:**
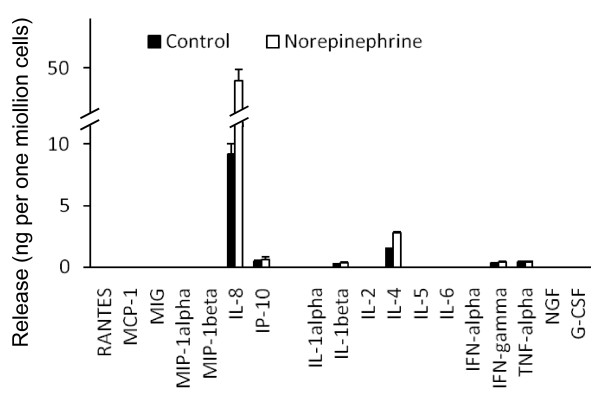
**Release of chemokines and cytokines by PC-3 human prostate carcinoma cells in response to norepinephrine**. The cells were incubated for 12 hours with 10 μM norepinephrine. The release of the shown substances in the culture medium was measured using a bead-based multiplex immunoassay and flow-cytometry according to the manufacturer's protocol (FlowCytomix, Bender MedSystems, Vienna, Austria). The graph shows mean values and standard deviation of three measurements. These are own unpublished data by Voss and Entschladen.

Catecholamines are metabolites of the amino acid tyrosine, namely dopamine, norepinephrine and epinephrine. Dopamine is produced in the brain and is released as a neurohormone with functions in the renal and hormonal regulation. Dopamine has also been implicated in schizophrenia and Parkinson's disease [13]. There are only few reports on the role of dopamine or the according receptors in tumor cell migration and metastasis formation, for which the following references might be most relevant with regard to this review's topic [14-16]. In contrast much more is known about the role of norepinephrine and epinephrine, the classical stress hormones. The main source of these neurotransmitters is the adrenal medulla. Norepinephrine and epinephrine are released in a stress reaction and cause an increase of the blood pressure, dilation of airways and glycogenolysis in the liver. Chronic stress has been implicated in tumor progression as early as 1926 [17], and several lines of epidemiological [18,19] and animal studies [20,21] support this view. Norepinephrine induces migratory activity of pancreatic [22], colonic [23], mammary [15], and prostate carcinoma cells [24]. With regard to the latter, these results have been confirmed by a mouse model showing that norepinephrine increases the formation of lymph node metastases by PC-3 human prostate carcinoma cells [25]. Furthermore, norepinephrine upregulates the release of vascular endothelial growth factor (VEGF) and interleukin-6 and -8 in melanoma cells pointing to a more aggressive potential of the cells [26]. With regard to the aforementioned PC-3 human prostate carcinoma cells, an upregulation of the release of interleukin-4 (1.5 ± 0.1 to 2.8 ± 0.1 ng per one million cells; p = 0.006) and of interleukin-8 (9.2 ± 0.8 to 48.9 ± 1.0 ng per one million cells; p < 0.001) was observed in response to norepinephrine, whereas some further chemokines and cytokines were released in minor amounts (Fig. 1; Voss and Entschladen, unpublished data).

Beta-blockers are clinically established drugs that are used in the treatment of hypertension. Their mechanism of action is to block beta-adrenergic receptors that are used by catecholamines to cause their regulatory effects on the blood pressure. These drugs are of certain interest in oncology, since there exist several lines of evidence that the above described function of catecholamines in tumor cell migration can be inhibited by beta-blockers [22,25,27], and beta-blockers might therefore work as anti-metastatic drugs. Interestingly, beta-blockers do not only counteract tumor cell migration and metastasis formation, but also cancer development *per se*; two epidemiological studies show that the incidence of cancer is reduced in patients that take beta-blockers [28,29].

### Gamma-aminobutyric acid

Gamma-aminobutyric acid (GABA) is a major inhibitory neurotransmitter of the brain, but has also an important function in the inhibition of glucagon secretion in the pancreatic alpha-cells. This neurotransmitter is discussed here, because it has been shown that it can counteract the promigratory effects of catecholamines. Engagement of GABA receptors inhibits the promigratory norepinephrine effect in pancreatic [30], colonic [31] and mammary carcinoma [15]. These effects are mediated by the metabotropic GABA_B_-receptor, for which baclofen is a selective agonist that is in clinical use for the treatment of epilepsy. Furthermore, systemic administration of baclofen in rats reduced carcinogenesis of gastric and colonic cancer [32,33]. Therefore, GABA-receptor agonists have been suggested to be introduced into cancer therapy [34].

### Inflammatory neurotransmitters

Chronic inflammatory processes can cause cancer, and conversely cancer can cause inflammatory processes. Regardless of what accounts for what, inflammation is clearly implicated in supporting tumor progression [35]. Although it is without doubt that the presence of leukocytes and pro-inflammatory cytokines and chemokines are the predominant factors for this inflammatory milieu in tumors [36], one might argue from several observations on inflammatory neurotransmitters that the nervous system can play a role in tumor progression as well. Furthermore, as shown in Fig. 1, certain non-inflammatory neurotransmitters can provoke the release of pro-inflammatory substances such as interleukin-8. However, histamine, bradykinin, calcitonin gene-related peptide (CGRP) and substance P are neurotransmitters that are known to have a direct regulatory function in inflammatory processes. Histamine is released by mast cells, and an accumulation of these cells around cutaneous tumors has multiple tumor-progressive effects [37]. Furthermore, histamine stimulates the migration of cervical carcinoma [38,39], as well as of epidermoid carcinoma and melanoma cells [39]. Bradykinin is a vasoactive nonapeptide, which has pro-inflammatory function and increases nociception. It has been described to enhance migration in bladder [40], chondrosarcoma [41] and prostate carcinoma cells [42]. With regard to the latter, this effect is specifically mediated by the bradykinin-1 receptor. This is in so far interesting, as the bradykinin-1 receptor was only detected in malignant lesions but not in normal prostate tissue [42]. In contrast to the constitutively expressed bradykinin-2 receptor, the bradykinin-1 receptor is underrepresented in normal tissue and upregulated during inflammation [43]. CGRP is abundantly present in the central nervous system, but also in nerve endings of peripheral nerves. In these neurons, it is frequently accompanied by norepinephrine and substance P. CGRP stimulates the invasive capacity of prostate cancer cell lines [44], but has no effect on the murine colon adenocarcinoma cell line Colon 26-L5 [45].

Substance P has multiple effects as neurotransmitter and neuro-modulator. It is involved in stress response and anxiety [46], and related psychological disorders such as schizophrenia and depression [47]. Furthermore, substance P plays a role as a modulator of nociception [48], and has various functions in inflammatory processes [49]. For example, it increases the cytokine release by macrophages under acute stress [50], and the chemokine production by neutrophil granulocytes [51]. It increases the cytotoxic activity of natural killer cells and at the same time reduces their migratory activity [52]. Furthermore, substance P induces migratory activity in cytotoxic T lymphocytes [52], and reduces the adhesion of these cells to vascular endothelium [53]. However, substance P plays a role not only as a direct mediator of inflammation but also communicates inflammatory processes in peripheral tissue to the brain, as has been extensively reviewed by Rosenkranz [54]. Very interestingly, in this review Rosenkranz discusses substance P as a mediator connecting psychological disorders and chronic inflammatory diseases. With regard to cancer and metastasis formation, substance P causes an increase of the basal-like human breast carcinoma cell line MDA-MB-468 [24], and plays a role in the development of bone marrow metastases in breast cancer and neuroblastoma [55]. Furthermore, substance P has an influence on tumor cell proliferation and angiogenesis, and therefore a blockade of the relevant receptor, NK-1, has been suggested as a new strategy in the treatment of cancer [56].

## Tumor innervation and the neuro-neoplastic synapse

How are neurotransmitters delivered to the tumor cells? Different mechanisms seem possible. Some of the neurotransmitters are systemically disseminated, e.g. the above discussed catecholamines. But others are only locally released by nerve endings, thus begging the question if tumors are actually innervated. There are currently only few reports available on this topic. Clinical observations on tumor innervation have been made concerning esophageal and cardiac carcinoma [57], as well as prostate cancer [58]. A further argument for tumor innervation is the fact that tumor cells release substances which are qualified to cause innervation. Tumor cells release axon guidance molecules [59], and other neurotrophic factors which have sometimes an overlapping function in tumor vascularization (neoangiogenesis) and lymph vessel development (lymphangiogenesis) [60]. For example, the nerve growth factor (NGF) has angiogenic effects [61,62], and in turn the vascular endothelial growth factor promotes not only angiogenesis but lymphangiogenesis and neurogenesis as well [62-64]. We thus argue that these three processes - neoangiogenesis, lymphangiogenesis, innervation - are likely to occur in concert. These three processes are not sole characteristics of tumors, but occur in any growing tissue in order to accomplish a proper connection of the new tissue to supply nourishment and superordinate regulation. However, sustained angiogenesis is one of the six hallmarks of cancer [65], and lymphangiogenesis is supposed to be of similar importance with regard to metastasis formation [66]. It is thus clear that these two processes support the growth and progression of a tumor. In contrast, it is not clear at first glance what kind of support might arise from tumor innervation, because the neuro-endocrine system is a superordinate regulatory system, which tumors evade. Two points are relevant here. The first point is, as we have discussed above, that neurotransmitters can increase cell migration and thus support metastasis formation. Such an interaction can occur in a neuro-neoplastic synapse which directly provides the neurotransmitters to the tumor cells [67]. However, such a synapse has yet only been described in functional aspects, by the observation of a mutual influence of signal substances that are released tumor cells and nerve cells on the respective other cell type as described herein. There is no morphological characterization so far. The second point is, that it is well described that tumor cells use nerve fibers as lines to migrate along, a phenomenon known as perineural invasion.

## Perineural invasion

Perineural invasion has been described for several types of cancer, as reviewed in [68]. However, the detailed molecular mechanisms, with which tumor cells interact with nerve cells are largely unknown. The embryonic adhesion molecule bystin has been shown to play a role in prostate cancer [69], and the neural cell adhesion molecule (N-CAM) is functionally implicated in various types of cancer, whereas the reports are conflicting with regard to whether the expression of the N-CAM correlates with perineural invasion or not. N-CAM expression has been reported in bile duct cancer [70], squamous cell carcinoma of head and neck [71], prostate cancer [72], and malignancies of the salivary gland [73]. In summary, although the phenomenon of perineural invasion is well recognized by clinicians in oncology since years, much less is known about its mechanisms in comparison to lymphogenous or hematogenous metastasis formation. Nevertheless, there is an increasing number of publications dealing with this issue, and perineural invasion may be regarded as a third way of metastasis formation independent of lymph or blood vessels [68].

## Concluding remarks

Several lines of evidence exist that tumor cells interact with the nervous system and that they are capable of responding to its soluble signaling molecules. Different from its role for normal tissue, the nervous system does not have the function of a superordinate regulatory organ for cancer cells, but can still support metastasis in at least two ways. Firstly, neurotransmitters can directly induce cell migration or regulate other parts of the metastasic multi-step process. Secondly, tumor cells can use nerve fibers as routes for invasion and emigration from the primary tumors. The latter is of course experimentally difficult to handle, and there are only few methods established on this. One of the most advanced methods is probably that used by Ayala *et al*., who co-cultured dorsal root ganglia from mice with tumor cells [74].

## List of abbreviations

CGRP: calcitonin gene-related peptide; GABA: gamma-aminobutyric acid; GPCR: G protein-coupled receptor; SDF-1: stromal cell-derived factor-1; N-CAM: neural cell adhesion molecule; NGF: nerve growth factor; VEGF: vascular endothelial growth factor.

## Competing interests

The authors declare that they have no competing interests.

## Authors' contributions

Both authors contributed equally. Both authors read and approved the final manuscript.
